# A cost-effectiveness study of caesarean-section deliveries by clinical officers, general practitioners and obstetricians in Burkina Faso

**DOI:** 10.1186/1478-4491-7-34

**Published:** 2009-04-16

**Authors:** Sennen H Hounton, David Newlands, Nicolas Meda, Vincent De Brouwere

**Affiliations:** 1 Department of HIV/AIDS & Reproductive Health, Centre MURAZ, Bobo-Dioulasso, Burkina Faso; 2 University of Aberdeen, Aberdeen, UK; 3 Institute of Tropical Medicine, Antwerp, Belgium; 4 Institut de Recherche pour le Développement, Rabat, Morocco; 5 Institut National d'Administration Sanitaire, Rabat, Morocco

## Abstract

**Background:**

The aim of this paper was to evaluate the effectiveness and cost-effectiveness of alternative training strategies for increasing access to emergency obstetric care in Burkina Faso.

**Methods:**

Case extraction forms were used to record data on 2305 caesarean sections performed in 2004 and 2005 in hospitals in six out of the 13 health regions of Burkina Faso. Main effectiveness outcomes were mothers' and newborns' case fatality rates. The costs of performing caesarean sections were estimated from a health system perspective and Incremental Cost-Effectiveness Ratios were computed using the newborn case fatality rates.

**Results:**

Overall, case mixes per provider were comparable. Newborn case fatality rates (per thousand) varied significantly among obstetricians, general practitioners and clinical officers, at 99, 125 and 198, respectively. The estimated average cost per averted newborn death (x 1000 live births) for an obstetrician-led team compared to a general practitioner-led team was 11 757 international dollars, and for a general practitioner-led team compared to a clinical officer-led team it was 200 international dollars. Training of general practitioners appears therefore to be both effective and cost-effective in the short run. Clinical officers are associated with a high newborn case fatality rate.

**Conclusion:**

Training substitutes is a viable option to increase access to life-saving operations in district hospitals. The high newborn case fatality rate among clinical officers could be addressed by a refresher course and closer supervision. These findings may assist in addressing supply shortages of skilled health personnel in sub-Saharan Africa.

## Background

Maternal and neonatal mortality remain unacceptably high in the developing world [1]. The risk of dying from pregnancy-related causes in the poorest countries is 250 times that of the richest countries [2].

Several strategies have been developed to significantly reduce these avoidable deaths, particularly the promotion of access to and uptake of life-saving interventions [3]. These life-saving interventions are contingent on the availability of skilled human resources. But several developing countries are faced with scarce and inadequate coverage of skilled health personnel.

In Burkina Faso, as in many other developing countries, doctors are concentrated in urban areas and there is high turnover (internal mobility towards international organizations and external brain drain) of skilled health professionals [4]. In most sub-Saharan countries, the use of substitute health workers began as a temporary measure while more doctors were trained, but has become a permanent strategy in the face of a crisis in human resources for health. Given the current pace of training skilled health professionals, it is unlikely that these countries will meet adequate and equitable ratios per population in the foreseeable future.

Subsequently, several countries have embarked on alternatives of training mid-level health professionals to address the shortage of skilled health professionals and in an attempt to contain costs [5,6]. Examples are clinical officers in Burkina Faso (*attachés de santé en chirurgie*), the Democratic Republic of the Congo, Mali, Mozambique, Niger, Tanzania and Zambia.

In Burkina Faso, a six-month special curriculum was designed to train district medical officers in emergency surgery (*chirurgie essentielle*: essential surgery). In Niger, a similar curriculum (*médecin capacitaire*) has been designed as a one-year course for general practitioners. Graduates can be directly admitted to the second year of training of obstetricians or surgeons.

Another similar training programme has been developed in Mali (*médecin à tendance chirurgicale*). Using mid-level cadres as substitutes for obstetricians or surgeons appears to be less costly and to help improve coverage of emergency obstetric care in rural areas [7-9].

Although much has been written about the effectiveness and cost of care of mid-level cadres [5-8], very few studies have looked at the cost-effectiveness of these alternative strategies in reducing maternal and neonatal deaths [10]. This study sought to contribute to the debate about the delegation of surgical tasks and use of substitutes by comparing the effectiveness and cost-effectiveness of caesarean-section deliveries in Burkina Faso by clinical officers – registered nurses with an additional two years' training in surgery (*attachés de santé en chirurgie*) – general practitioners trained in essential surgery and obstetricians in reducing maternal and neonatal mortality.

## Methods

### The context

The study was conducted in Burkina Faso, one of the poorest countries in the world [11], with a 23% adult literacy rate and a high maternal mortality ratio estimated as ranging from 484 per 100 000 live births [12] to 700 per 100 000 live births [13]. In 2005, the average ratios of specialists, general practitioners and midwives were 1 per 100 000, 1 per 30 000 and 1 per 25 000 inhabitants, respectively [14]. These figures were worse when considering subsets of these health personnel actually providing clinical care (and not involved in other activities).

To respond to the scarcity of skilled health providers, the government embarked in the early 1980s in training substitutes for skilled health professionals: training courses for auxiliary midwives (*accoucheuses auxiliaires*), male midwives (*maïeuticiens d'état*) and clinical officers (*attachés de santé*) were created.

In addition, a six-month training programme in essential surgery for medical doctors (*chirurgie essentielle or essential surgery*) was initiated in the early 1990s. Recent process evaluations of this latter programme [15,16] revealed a high turnover of trained doctors due to lack of reward in terms either of an increase in salary or of degree accreditation, a high rate of absenteeism due to lack of motivation, and competing administrative tasks.

### Study design, sampling and participants

The study was a retrospective, cross-sectional, facility-based survey. Data for 2004 and 2005 were collected from hospital records and patient case notes during the last quarter of 2007. Because of time and resource constraints, we decided to collect data from all public sector facilities providing caesarean sections in six of the 13 regions of the country (22 hospitals). These six regions were conveniently selected to account for major socioeconomic and cultural differences in the country. Participants in the study were providers of caesarean section, such as specialists (obstetricians or surgeons), trained general practitioners, clinical officers, support staff, policy-makers, and maternal and child health programme managers.

### Data collection

Data were collected by a survey team composed of two public health specialists and former district medical officers, three health economists, three sociologists and a midwife. Case extraction forms were used to systematically record data on caesarean sections from operating theatre books and delivery registers. The case extraction forms were pretested in a separate district hospital and pilot-tested before wider use in the selected study areas. Data were collected from each facility on number of caesarean sections, providers, referral status of cases, diagnosis at admission, interventions performed, survival outcomes for mothers and babies, postoperative complications (wound infection, haemorrhage, wound dehiscence), duration of operation, duration of postoperative inpatient days and type of anaesthetic.

Data were collected on the costs of putting together surgical teams to perform caesarean sections led by obstetricians, general practitioners or clinical officers. These data included annual salary, allowances, pension, training and deployment, and time spent on surgical tasks. In addition, data were collected from the university on the duration of training and from the Ministry of Health on the number of medical officers trained in emergency surgery at district level. Finally, interviews were conducted with providers and their surgical teams, policy-makers and programme managers on barriers and facilitators for the essential surgery training strategy.

### Costing assumptions

The main assumptions made in costing relate to the time of surgical team members and volume of caesarean sections, compared to other medical and surgical interventions, so as to determine the proportion of total costs attributable to caesarean sections (compared to other activities). The proportion of time spent by clinical officers on caesarean sections was approximated by assuming that clinical officers spend their entire time in surgical units and by dividing the number of caesarean sections by the total number of surgeries performed in 2006.

A self-administered time allocation form was used to approximate the proportion of time spent by trained physicians on caesarean sections compared to other activities (clinical and administrative). For non-surgical personnel (nurses, midwives, drivers, cooks, guards, etc.) and other hospital costs (mortuary, cleaning, motorcycles, etc.), we used an estimate of 2% to apportion costs to caesarean sections. Finally, the proportion of laboratory and operating theatre costs attributable to caesarean sections was estimated at each facility by dividing the volume of caesarean-related laboratory exams and operations by total laboratory exams and total life-saving surgeries for mothers, respectively.

The different discounting periods used are derived from the average times spent in public service after graduation by providers, assuming they remain in public service until retirement. As an example, a nurse could potentially work for 30 years after graduation, since, at the time the survey was conducted, retirement was at 55 years of age or after 30 years of public service. Clinical officers could potentially work 20 years, given that most clinical officers return to further training after an average eight to 10 years of nursing practice. The training of physicians in essential surgery was discounted over five years, because this is the minimum period of public service before they can seek specialized training. None of the trained physicians missed the opportunity to enhance their career by moving to public health training or a clinical speciality.

### Data analysis

Descriptive statistics were used to compute rates and ratios for each type of provider. Confidence intervals were constructed around each estimate. This was the preferred approach, since we wished to include all caesarean sections from district hospitals in the analysis. Case mixes by each type of provider were assessed by analysing the relationship of the key effectiveness measure with providers, adjusting for reported diagnosis and referral status (a proxy for the severity of cases).

We calculated cost estimates of strategies (surgical teams led by obstetricians, trained general practitioners or clinical officers at district hospitals) per selected outcomes, employing a health service perspective. The costing exercise was carried out for 2006. We estimated the costs of caesarean sections carried out by surgical teams led by each of the three providers, since we are seeking to compare strategies, the combinations of provider, surgical team and technical support. This approach was preferred because, apart from patients' clinical condition at admission, the outcomes of life-saving interventions depend on providers' skills and the presence of an adequate team, required drugs and functioning equipment.

Training costs were annualized, at a discount rate of 3%, so that they could be added to the other health human resource costs to derive a measure of the annual costs of putting together surgical teams to provide caesarean sections. The next step was to apply the appropriate proxies of time of surgical team members so as to determine the proportion of total costs attributable to caesarean sections, compared to other activities. Incremental cost-effectiveness ratios were computed by dividing the differences in average costs of putting in place a surgical team led by one type of provider compared to an alternative option by the differences in newborn case fatality rates associated with each option. Sensitivity analysis was conducted on the major cost categories.

We divided the average cost of putting in place an obstetrician/general practitioner/clinical officer-led surgical team by the average number of caesarean sections performed at the district hospitals in 2006. We considered this latter measure the closest approximation of average throughput across the whole country. Implicit in our analysis was that variable costs would be the same for an obstetrician-led team, a general practitioner-led team, and a clinical officer-led team. In fact, the main element of variable costs is the cost of kits and this cost is borne by patients (although subsidized by the government since October 2006) and therefore not included in our costing, which is from a health service perspective.

The costing exercise was conducted in West African CFA (*Communauté financière d'Afrique *– Financial Community of Africa) francs, the currency of Burkina Faso. Key results were then translated into international dollars, which are United States dollars adjusted for differential purchasing power. In 2006, one international dollar equalled 181 CFA.

## Results

### Case profile and effectiveness of caesarean-section deliveries

Table 1 describes the profile of 2305 cases, postoperative complications and the duration of postoperative hospital stay, by type of provider. It is important to clarify here that the clinical diagnoses in Table 1 are not indications for caesarean sections, but the diagnoses as indicated in records after surgery. There were no significant differences in maternal age, the clinical indications for caesarean delivery and postoperative complications such as haemorrhage, wound infection and wound dehiscence (results not shown).

**Table 1:** Profile of caesarean deliveries by type of provider, Burkina Faso, 2004–2005

**Type of providers** **Characteristics**	**Obstetricians** N = 1020*	**Trained doctors** N = 552*	**Clinical officers** N = 733*
**Place (% within facilities)**			
National hospitals	53	3	0
Regional hospitals	17	2	41
District hospitals	30	95	59

**District hospitals (% rural versus urban)**			
Urban**	86	5	27
Rural	14	95	73

**Mother's age**			
Median (IQR)	25 (10)	24 (12)	25 (12)
**Indication of surgical procedure **(%)			
Saving mother's life	74	75	81
Saving baby's life	24	21	17
Other	2	4	2
**Mothers' reported conditions **(%)			
Obstructive labour	39	51	53
Ruptured uterus	11	7	11
Eclampsia	7	1	2
Haemorrhage	5	6	6
Other	38	35	28
**Type of anaesthesia **(%)			
General	81	30	59
Spinal anaesthesia	19	70	41
**Referral status **(%)			
Referred from other facilities	85	77	71
Referred by provider to higher level facility	15	23	29
**Duration of caesarean-section **(minutes)			
Mean (SD)	46 (20)	57 (27)	53 (23)
**Duration of post operative hospital stay **(days)			
Mean (SD)	6 (5)	9 (5)	9 (6)
**Post operative complications **(/000)			
Haemorrhage	15	18	20
Wound infection	11	4	14
Wound dehiscence	4	0	1

* Counts were for a complete year at district hospitals.

** Only three districts (all urban) had obstetricians at the time of the survey, and only one (District Secteur 30) was fully functional with regard to obstetric surgery. A quarter of the caesarean delivery case notes were extracted by the data collection team.

Clinical officers and trained general practitioners perform most emergency obstetric surgery at district hospitals, and obstetricians and surgeons practise in the three urban district hospitals and at the regional and national levels. On average, 50% of cases dealt with at district hospitals by clinical officers and trained general practitioners are obstructive labour, compared with less than 40% for obstetricians.

There was a significant difference in the percentage of eclampsia, though the overall proportion of such cases is small (6%). Similarly, there was a 20% shorter duration for the operation and a 30% shorter duration of postoperative hospital stay with obstetricians. Obstetricians were also more associated with referred cases (a proxy for the severity of cases and delay in gaining access to care), but this difference is not statistically significant (results not shown).

**Figure 1:**
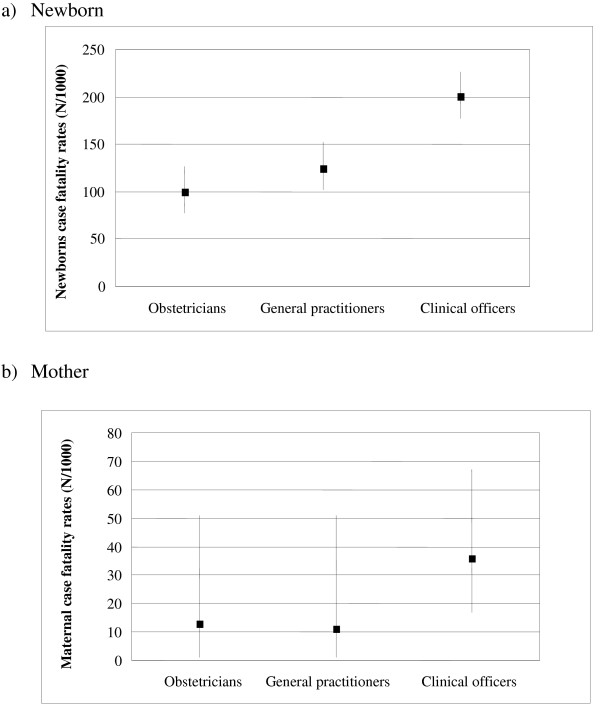
**Case fatality rates of caesarean deliveries by provider, district hospitals, Burkina Faso, 2004–2005**.

Figure 1 presents the case fatality rates (CFR) for newborns and mothers after caesarean deliveries, by type of provider. The CFR for newborns was significantly higher for clinical officers (198 per 1000 compared, to 99 for obstetricians and 125 for trained general practitioners). Similarly, there seems to be a difference in CFR of mothers, with higher mortality associated with clinical officers, although statistical significance was not reached.

### Facilitators and barriers to the essential surgery strategy

Analysis of interviews with obstetricians, trained doctors, clinical officers, surgical aides and policy-makers revealed that the delegation of surgical skills to mid-level cadres (trained doctors, clinical officers) was necessary and has been useful in increasing access to life-saving interventions for women and babies. However, most trained doctors are often out of hospital for administrative duties and tend to move into public health training on average five years after their essential surgery training. The regulation is that any public servant can seek continuing education five years after being hired. According to the interviews, the reasons for rapid turnover were the absence of incentives to remain in post (no supervision, no increase in salary and no clear career path).

### Costing caesarean sections by type of provider

Table 2 shows the annual training and deployment costs of providers. The annual training cost of an obstetrician was estimated at 1.49 million CFA, or 8231 international dollars, 30% higher than the training costs of a trained doctor (1.04 million CFA, 5747 international dollars) and over 80% higher than the training costs of a clinical officer (0.27 million CFA, 1480 international dollars).

**Table 2:** Annual training and deployment costs of providers of caesarean deliveries, Burkina Faso, 2006

**Clinical officers**	**Costs (CFA)**
Basic clinical officers training (discounted over 30 years) cost/resident^1^	73 127

Clinical officers training costs (discounted over 20 years)	194 768

Total clinical officers training costs	267 895

Enhanced clinical officers training*	

Discounted basic clinical officers training (discounted over 20 years) cost/resident^1^	73 127

Clinical officers training costs (discounted over 20 years)	194 768

Refresher course** (discounted over 2 years) *	313 589

Total enhanced clinical officers training costs	581 484

**Trained doctors in essential surgery**	

Basic training (discounted over 30 years) cost per doctor	135 171

Surgery training costs (discounted over 5 years)^5^	904 983

Total essential surgery training costs	1 040 153

Enhanced essential surgery training*	

Basic training (discounted over 30 years) cost per doctor	135 171

Enhanced surgery training costs (discounted over 15 years)^5^	1 296 608

Total enhanced surgery training costs	1 431 779

**Specialists (obstetricians and surgeons)**	

Basic training (discounted over 30 years) cost per doctor	135 171

Obstetricians' training costs (discounted over 20 years)^7^	1 354 650

Total obstetricians' training costs	1 489 821

^1^From nursing school budget, 2005, numbers of students (all residents, all majors, % clinical officers), national contest for clinical officers, discounted over 30 years

^2 ^Source: Human resources division, health personal salaries.

^3^Discounted over 20 years

^4^From University of Ouagadougou budget, School of Health Sciences (% of medical students among all students), national contest, discounted over 30 years

^5^Source: Ministry of Health, discounted over five years (average duration of practice of essential surgery)

^6 ^From University of Ouagadougou budget, School of Health Sciences (% of medical students among all students), national contest

^7^Discounted over 20 years (estimated duration of practice of obstetrician)

* See Table 4

** Estimated refresher course and supervision costs, discounted over two years (clinical officers to attend two month refresher course every two years)

Table 3 shows the annual costs of putting together surgical teams to provide caesarean sections for each of the district hospitals. The annual cost of each surgical team varied significantly by type of provider and among district hospitals for a specific provider. As an illustration, the annual cost of an obstetrician-led team was 1 213 239 CFA or 6703 international dollars in Pissy district hospital, 38 times the cost in Secteur 30 district hospital (31 696 CFA, or 175 international dollars). These large economies of scale are also illustrated by the significant difference in annual costs of general practitioner-led teams between Boulsa and Bogande, two remote, rural district hospitals.

**Table 3:** Annual costs of caesarean deliveries by type of provider teams at district hospitals, Burkina Faso, 2006

	**Cost of provider-led surgical team**			
		
**District hospitals**	**Obstetricians**	**General practitioners**	**Clinical officers**	**Number of** c-sections*
Barsalogo	-	6 078 044	5 691 552	37

Bogande	-	3 970 104	3 604 590	56

Boromo	-	5 023 738	4 565 343	144

Boulsa	-	2 253 927	1 889 286	4

Diebougou	-	1 693 474	1 301 525	51

Diapaga	-	1 829 962	1 466 812	81

Houndé	-	3 328 228	2 958 590	112

Kongoussi	-	4 443 142	4 079 992	27

Kossodo**	1 142 510	616 192	-	11

Nouna	-	3 365 654	1 457 758	42

Orodara	-	3 595 155	3 041 070	89

Pama	-	1 895 451	1 513 389	12

Pissy**	3 639 716	2 576 760	-	3

Secteur 30**	20 950 981	-	-	661

Solenzo	-	3 855 850	2 893 375	87

Tougan	-	7 482 383	7 058 708	61

**Mean**	**8 577 736**	**3 466 938**	**3 222 433**	**92.375**

* From a prospective data collection on caesarean-sections per district hospital, during 2006

** Urban district hospitals

The upper part of Table 4 shows the total cost to the health system of caesarean sections by surgical team. The average total cost of obstetrician-led teams was 8.58 million CFA, or 47391 international dollars, the average of the three district hospitals where there are obstetricians. The corresponding estimate of general practitioner-led teams was 3.47 million CFA, or 19 154 international dollars, the average of the 15 district hospitals where there are trained general practitioners. The average total cost of clinical officer-led teams was 3.22 million CFA, or 17 803 international dollars, the average of the 13 district hospitals where clinical officers conducted caesarean sections.

**Table 4:** Incremental cost-effectiveness ratios of caesarean deliveries by providers' teams, district hospitals, Burkina Faso, 2006

**Providers** **(Surgical teams led by...)**	**Total costs of surgical team**	**Cost per c-section, CFA**	**Newborns CFR** **(/1000 c-sections)**
Obstetricians (O)	8 577 736	92 858	99

Trained doctors (D)	3 466 938	37 531	125

Clinical officers (CO)	3 222 433	34 884	198

Incremental Cost-Effectiveness Ratio (ICER) = incremental cost of performing 1000 c-sections/incremental gain of newborns' lives per 1000 c-sections			

ICER from (D) to (O) = (92.858 mi-37.531 mi)/(125-99)			2 127 962

ICER from (CO) to (O) = (92.858 mi-34.884 mi)/(198-99)			585 596

ICER from (CO) to (D) = (37.531 mi-34.884 mi)/(198-125)			36 260

The large difference between the cost of obstetrician-led teams and general practitioner-led teams is due partly to the costs of training and remuneration of obstetricians but mostly to the greater support available, in terms of other personnel, at the urban district hospitals where obstetricians are to be found. In contrast, the (smaller) difference between the cost of general practitioner-led teams and clinical officer-led teams is due largely to the greater costs of training and remuneration of general practitioners.

We estimated the average cost per caesarean section to be 92 858 CFA (513 international dollars) per caesarean section conducted by obstetricians, 37 531 CFA (207 international dollars) for general practitioners and 34 884 CFA (193 international dollars) for clinical officers. These are all large figures, but they reflect the small number of caesarean sections performed at district hospitals – indeed all hospitals – in Burkina Faso. If the average number of caesarean sections performed by any of the surgical teams increased, the average cost per caesarean section would fall correspondingly. Very significant economies of scale could be achieved within the existing capacity of district hospitals. Indeed, if the average number of caesarean sections performed at district hospitals were to double, we would expect the cost per caesarean section to halve.

### Cost-effectiveness analysis

The lower part of Table 4 presents the Incremental Cost Effectiveness Ratio (ICER) of adverse outcomes for newborns after a caesarean delivery, by type of provider. These figures can be interpreted as the additional cost of saving a newborn's life, moving from one provider to another. For example, subtracting the average cost of 1000 caesarean sections performed by general practitioners from that performed by obstetricians (92 858 million minus 37 531 million CFA) and dividing by the difference in performance (125 minus 99 newborn CFR per 1000 caesarean sections) gives an ICER of 2 127 962 CFA. This means that the cost of avoiding one additional newborn death when 1000 caesarean deliveries are performed by an obstetrician instead of a trained doctor is 2 127 962 CFA, or 11 757 international dollars. The ICER of caesarean sections performed by an obstetrician rather than a clinical officer is 585 596 CFA, or 3235 international dollars, and of caesarean sections performed by a general practitioner rather than a clinical officer, 36 260 CFA, or 200 international dollars.

There is no agreed threshold value above which an option would be considered unacceptable. Nevertheless, while we may not be able to decisively reject any option as not being cost-effective, taken together our results are indicative of the effectiveness and cost-effectiveness of trained general practitioners.

Table 5 presents results of a simple modelling of the enhanced training and consequent better performance of general practitioners and of clinical officers. In addition, we assumed that turnover would be reduced and more career development opportunities provided for general practitioners. A two-year training programme would improve quality of care and would qualify doctors for an increase in salary within Burkina Faso's public health system. A benefit package of such enhanced training would address barriers to current essential surgery training and would include management responsibilities at district level, allowances for living conditions in remote areas and a career path opportunity to directly join the training class of obstetricians or surgeons with the automatic validation of the first year of study.

**Table 5:** Incremental cost-effectiveness ratios of caesarean deliveries by providers' teams, enhanced strategies*, district hospitals, Burkina Faso, 2006

**Providers** **(Surgical teams led by...)**	**Total costs of surgical team**	**Cost per c-section (CFA)**	**Newborns CFR** **(/1000 c-sections)**
Obstetricians (O)	8 577 736	92 858	99

Trained doctors (D)	4 205 141	45 523	112**

Clinical officers (CO)	3 796 782	41 102	161.5***

Incremental Cost-Effectiveness Ratio (ICER) = incremental cost of performing 1000 c-sections/incremental gain of newborns' lives per 1000 c-sections			

ICER from (D) to (O) = (92.858 mi-45.523 mi)/(112-99)			3 641 154

ICER from (CO) to (O) = (92.858 mi-41.102 mi)/(161.5-99)			828 096

ICER from (CO) to (D) = (45.523 mi-41.102 mi)/(161.5-112)			89 313

* Enhanced strategies = enhanced essential surgery and enhanced clinical officers

"Enhanced essential surgery" = two years' degree-seeking training, salary incidence, incentives (management at district level, allowances for living conditions in remote areas, and possibility to directly join obstetricians' or surgeons' training class with validation of first year); assumptions of 15 years' practice, and advantage of not losing critical life saving to public health training and practice

"Enhanced clinical officers" = current clinical officers subjected every two years to a refresher course coupled with an effective supervision programme

** Hypothetical figure of outcomes of "enhanced essential surgery" after two year training programme

*** Hypothetical figure of outcomes of "enhanced clinical officers" after refresher courses and effective supervision

The costs of putting in place a general practitioner-led surgical team rise as a consequence of the increased training period, from 3.47 million CFA, or 19 154 international dollars, to 4.21 million CFA, or 23 233 international dollars. Performance can be assumed to improve as a result of greater skills and commitment, but there is no way to determine what the improvement will be. We assume that half the previous gap in performance between obstetricians and trained general practitioners would be closed – that the newborn CFR (per 1000 caesarean sections) for trained general practitioners would fall from 125 to 112. We now assume that trained doctors have an incentive to remain in service performing caesarean sections for longer, and that they do so for 15 years (as opposed to five years previously).

For clinical officers, we model an enhancement of their training, consisting of a refresher course every two years and more effective supervision. The costs of putting in place a clinical officer-led surgical team rise as a consequence, from 3.22 million CFA, or 17 803 international dollars, to 3.80 million CFA, or 20 977 international dollars. Again, performance can be assumed to improve but we cannot say what the improvement would be. However, following the same approach as before, we assume that half the previous gap in performance between trained general practitioners and clinical officers would be closed – that the newborn CFR (per 1000 caesarean sections) for clinical officers would fall from 198 to 161,5.

As can be seen from Table 5, all the Incremental Cost-Effectiveness Ratios (ICERs) increase. The most important change is the ICER from general practitioners to obstetricians, which increases from 2 127 962 CFA, or 11 757 international dollars, to 3 641 154 CFA, or 20 117 international dollars. This is further evidence of the cost-effectiveness of trained general practitioners.

## Discussion

Within the limits of comparability of the different configuration of surgical teams led by each type of provider, it appears that both training clinical officers and general practitioners, particularly the latter, are viable options to increasing coverage of emergency obstetric care in district hospitals. Training of general practitioners appeared effective and cost-effective. Levels of performance could be increased further by improving the supervision of clinical officers and providing trained doctors with stronger incentives, such as better career opportunities.

Despite the importance of these findings, some limitations of the study should be acknowledged. We sought to compare outcomes of caesarean sections among three different types of surgical team, led by an obstetrician, a trained general practitioner or a clinical officer. This was deemed necessary because it is technically difficult to assume comparability of scope, skills and leadership among the providers and it is also difficult to use emergency obstetric care, a more complex entity, as an output without taking into account the detailed case mix.

Our approach therefore was to compare outputs of caesarean sections at district hospital level. The construction and equipment of district hospitals are standardized in Burkina Faso [17], albeit urban district hospitals are better equipped.

The ability to perform caesarean sections was used as a proxy for the ability to perform life-saving obstetric surgery, an assumption that may be flawed because of differentials in skills to perform life-saving interventions. Also, the reliability of our main effectiveness measure, newborn case fatality rates, suffered from lack of precision on the timing of deaths, which would have been useful in associating deaths with surgical teams or monitoring of the labour. We assumed that deaths of newborns following caesarean sections are associated with the management of cases by health teams at hospital level, which may not always be the case.

Finally our operational approach to assess the comparability of cases by type of provider was to adjust for reported diagnosis after surgery and by referral status (a proxy for delay in accessing care and severity). The reliability of this approach may be limited for a retrospective study.

The duration of operations and postoperative hospital stay was 10% and 30% shorter, respectively, with obstetricians, probably reflecting their better skills and practices in obstetrics. Also, obstetricians were more associated with referred cases and eclampsia, because more severe cases are referred to higher-level facilities where obstetricians and surgeons are more likely to practise. The observed differences reflect more the differences in resources available at each level of the health system. As an illustration, providers will rely on spinal anaesthesia in remote areas that lack a resuscitation system and intensive care unit, while at regional and national hospitals the preferred means of induction will be general anaesthesia.

Beyond the issue of comparability of cases, a more rigorous approach would have taken into account indication and relevance for caesarean sections, delay in reaching the decision to operate, delay between the decision to operate and actual interventions, and the clinical condition of mothers and unborn babies on arrival at hospital [18]. Unfortunately, retrospective records are deficient and a prospective data collection was beyond the scope of this study. It is therefore difficult to reliably attribute adverse outcomes to configurations of providers. Any inferences should thus be made with caution.

For the foreseeable future, these categories of health professionals will remain the only available resources in remote and rural settings in Burkina Faso and it is crucial that quality of care be ensured. Experience from other settings of using substitutes for skilled health professionals led to the conclusion that these strategies may be the only options to increase coverage of life-saving procedures in the short or medium term [7-9]. However, given the scarcity of resources, policy-makers should embark on the most cost-effective strategies and therefore these strategies should be weighed against health gain in terms of preventing deaths of mothers and newborns.

The overall CFRs are too high, irrespective of providers of caesarean sections, if the country is to meet the targets of Millennium Development Goals Four (to reduce child mortality) and Five (to improve maternal health). Vaz et al. have reported similar rates (0.4%) of postoperative maternal mortality in emergency interventions by medical assistants in Mozambique [5]. Unlike the experience of Mozambique and Malawi with clinical officers [7,10], postoperative maternal and neonatal mortality rates in our study were not comparable to those of trained general practitioners or obstetricians. The excess of mortality of newborns and mothers with clinical officers indicates the need for refresher courses and better supervision for clinical officers if maternity care is to be improved.

## Conclusion

Based on the observed level of performance of providers of caesarean sections, trained general practitioners appear to be the most cost-effective option for increasing coverage of maternal life-saving interventions. Their cost-effectiveness could be further increased if they are provided with better career paths and if incentives for general practitioners resulted in further improvements in their performance (Table 5). Given that Burkina Faso will not have the required rates of skilled health professionals in the short run, delegation of surgical tasks from specialists to middle-level health substitutes appears necessary and effective. These findings may assist other sub-Saharan African countries to address supply shortages of skilled health personnel.

## Competing interests

The authors declare that they have no competing interests.

## Authors' contributions

SHH was the principal investigator of the grant. He led data collection and data management; performed the analysis; drafted and finalized the manuscript. DN contributed to the conception of the grant and assisted with study design; performed data analysis; and contributed to the interpretation and writing of the manuscript. NM contributed to the conception of the grant; assisted with study design, data analysis and interpretation and intellectual content of the manuscript. VDB contributed to the conception of the grant, assisted with data analysis and interpretation and writing of the manuscript.
